# A proposed numerical method for absolute efficiency calibration of *α*-spectrometers and its application for activity calculation

**DOI:** 10.1016/j.heliyon.2024.e28498

**Published:** 2024-03-25

**Authors:** K.M. El-Kourghly, W. El-Gammal, Mohamed M. Damoom, Abdulsalam M. Alhawsawi, M. Abdelati, H.I. Khedr, R. Abouzaid

**Affiliations:** a Nuclear Safeguards and Physical Protection Department, Egyptian Atomic Energy Authority (EAEA), Cairo, Egypt; b Nuclear Engineering Department, King Abdulaziz University, Jeddah, 21589, Saudi Arabia; c Center for Training & Radiation Protection, King Abdulaziz University, Jeddah, 21589, Saudi Arabia; d Fuel Cycle Safety Department, Egyptian Atomic Energy Authority (EAEA), Cairo, Egypt

**Keywords:** Alpha spectrometers, Absolute method, Analytical-numerical, MCNP, Solid angle

## Abstract

In this work, an absolute method to calibrate an *α*-spectrometer is proposed taking into account the Source-to-Detector, and lateral distances due to eccentric source distribution. An analytical formula to calibrate an *α*-spectrometer is derived and the Simpson's integration method was utilized to solve these equations in its integral form numerically using a written C computer code. The general Monte Carlo N-particle code, MCNP as well as experimental measurements for some standard *α*-emitters are used to benchmark the proposed method. An agreement was found between the efficiency results calculated by MC and the proposed method with a maximum relative difference of about 0.5%. While, experimental measurement of *α*-emitters activity employing the proposed method differs by about 1.65% from the certified values. Accounting for the man made error allows to accurately quantify the assayed sample. Therefore, the inaccuracy in the efficiency results due to non-accurate inputs pertained to the source, and detector radii, Source–Detector distances, and eccentric source distribution are investigated in the Source-to-Detector distance range of (4 to 44 mm). The results show that a difference of ±1% in the detector radius, and Source-to-Detector distance than the normal values yields a relative difference of about ±2%, while a difference of ±50% in the source radius or source lateral distance from detector symmetry axis could only yields inaccuracy of less than ±3% in the efficiency results.

## 1 Introduction

Alpha spectrometry is widely used in the field of Nuclear Safeguards (NSG) as a radioanalytical technique. Mainly because of its high sensitivity, the analysis of comparatively small sample size is feasible. The number of nuclei that may be reliably determined by *α*-spectrometry is quite large. It covers almost all *α*-emitting nuclides with long half-lives relative to the sample preparation time [1]. Within the field of NSGs *α*-spectrometry may well be used for nuclear materials (U, Pu, and Th) characterization and verification including, the determination of uranium and plutonium isotopic ratios, and isotopic mass and activity [2], [3], [4], [5].

The isotopic activity of *α*-particle emitting radionuclides is determined employing either relative or absolute efficiency calibration methods. Within the relative method, a standard alpha sources with known activity and identical geometry to the assayed sample (same size of the active source area, same distance from detector) are used to determine the detector efficiency experimentally. It could be achieved by calculating the ratio of the measured net count rates to the certified emission rate [1], [6], [7].

Relative method based on standard sources is used for efficiency calibration of *α*-spectrometers by Vioque et al. [8], Bochud et al. [9], Chamizo et al. [10], Calin et al. [11] to estimate the activity of *α*-emitters in environmental measurements [8], [9], [10], and quality assurance [11] aspects. They mentioned that, they have got a relative error of about 1.5%.

The relative method provides an accurate efficiency calibration however, standard calibration sources rarely have the same geometry as the assayed sample [12]. The discrepancy in standard and sample geometry causes a significant inaccuracy in the counting efficiency. Consequently, absolute methods based on mathematical efficiency calibration [13], [14], [15] could be considered whenever calibration standards are failed.

The absolute *α*-counting efficiency $\varepsilon_{a}$ is defined as the number of pulses recorded to the number of alpha particles emitted by the source [6], [7].

One of the *α*-detector characteristics is that their intrinsic efficiency is close to unity due to the much greater mass of the *α*-particle. Unless the detector thickness is lower than the range of *α*-radiation in the detector material, all *α*-particles that reach the active volume of the detector are counted which makes the absolute efficiency estimation is limited to the geometry factor determination. It can be calculated if the geometry parameters such as source and detector areas and Source-to-Detector (S–D) distance are known exactly.

The key point for calculating the geometrical efficiency is the computation of the Solid Angle (SA). In this regard, SA determination has been addressed in many publications either in *α*-spectrometry or pertaining to analogous problems in other research fields. The SA of disk detector subtended by a coaxial disk source is calculated by Gardner et al. [16], Ruby [17], Pommé et al. [18], El-Gammal et al. [19], El-Kourghly et al. [20], Carrillo [21], Díaz et al. [22] using either analytical calculation, or the MC method.

It can be seen from the above review that the absolute efficiency of the *α*-spectrometer is based on experimental measurements that must provide an identical standard or a mathematical modeling that specifically focuses on the coaxial S–D configuration. This study aims to propose a numerical technique for estimating the absolute efficiency of *α*-detector in different S–D arrangements (including axial and non-axial S–D configurations). The method is based on theoretical calculations; therefore, standard sources are not necessary. The general Monte Carlo N-Particle, MCNP Code calculations and experimental measurements are performed to benchmark the proposed method.

## 2 Methods description

The proposed numerical method is achieved through two main steps: First, analytical formulas to determine the absolute counting efficiency of an *α*-detector are derived at different S–D configurations and a computer code is written using a C programming language to solve the derived formula in its integral form numerically. In the second step, the MCNP code calculations, as well as experimental measurements, are used to evaluate the proposed method.

### 2.1 Analytical calculations

The calculated activity *A* $\left(s^{-1}\right)$ of a specific *α*-energy line could be expressed as

(1) $$A=\frac{C_{R}}{\varepsilon_{a}\;\alpha },$$

where,

$C_{R}$: is the net count rate $\left(s^{-1}\right)$ for a given *α*-energy line with emission probability of *α*,

$\varepsilon_{a}$: is the absolute detector efficiency, that could be expressed as,

(2) $$\varepsilon_{a}=\varepsilon_{i}\;\Omega_{f}\;f_{d}\;f_{p},$$

where,

$\varepsilon_{i}$: is the intrinsic detector efficiency,

$\Omega_{f}=\frac{\Omega }{4\pi }$: is the fractional solid angle of the source with respect to the detector (total radiation fraction that passes through the detector surface),

$f_{c}=f_{d}\;f_{p}$: is the correction factors due to the dead-time and pileup effects.

The Solid angle Ω of the detector with respect to the source is defined as [1], [6], [7];

(3) $$\Omega =\int_{\theta }\int_{\phi }d\theta d\phi \;\sin\theta ,$$

where, *θ* (0−π) is the polar angle around z-axis and *ϕ* (0−2π) is the azimuthal angle on the x–y plane. The linear stopping power of *α*-particles through the detector material is very high, thus the detector intrinsic efficiency $\varepsilon_{i}$ is approximately 100%. Also, the correction factors due to electronic losses are corrected by reducing the detector dead-time. Thus, the detector absolute efficiency is mainly depend on the S–D geometrical configuration or the geometric efficiency $\varepsilon_{G}$ that takes the following form [1], [6], [7];

(4) $$\varepsilon_{G}=\frac{1}{4\pi }\int_{0}^{2\pi }\int_{0}^{\pi }d\theta d\phi \;\sin\theta .$$

**Figure 1:**
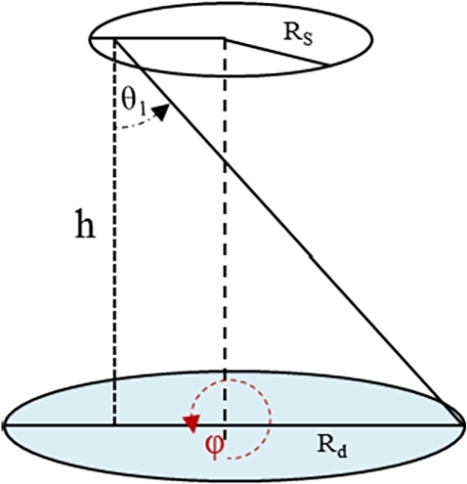
Geometrical arrangement of the symmetrically located disk source and the disk detector.

Fig. 1 shows a 3D representation of axial *α*-disk source located symmetrically and ⊥ the axis of the detector. The source has radius $R_{s}$ is situated at a distance *h* from the front facet of the detector of radius $R_{d}$. From the notation given in Fig. 1, the general equation that could be used to calculate the geometrical efficiency $\varepsilon_{G}$ of a parallel axial and non-axial S–D configurations at different S–D distances could be written as follows [19], [20];

(5) $$\varepsilon_{G}=\frac{1}{\pi R_{s}^{2}}\sum_{n_{i}}^{n_{m}}\int_{\rho_{n,i}(1)}^{\rho_{n,m}(2)}\int_{\phi_{n,i}(1)}^{\phi_{n,m}(2)}\int_{\theta_{n,i}(1)}^{\theta_{n,m}(2)}\;d\theta \;d\phi \;dr_{s}\;r_{s}\;\sin\theta ,$$

where, *n*: is the configuration number, n=1,2,…,4,

*i*: represents the number of terms of integration for the configuration number *n*, i=1,2,3,…,m term and

$\rho_{n}$: is the lateral distance from the detector central axis $(\rho )=R_{s}+l$ and *l* is the lateral distance between the central axes of the detector the source of configuration number *n*.

The integration limits for the polar *θ* and azimuthal *ϕ* angles, and disk source dimensions are presented in Appendices A and B.

### 2.2 MC simulation

The MCNP calculations are performed to benchmark the derived equations. Thus, the geometrical efficiency is calculated using the MCNPX [23], that contains F1 surface current tally (i.e., geometrical efficiency). Void geometry is simulated considering two circular surfaces ensembled inside a vacuum chamber with axial and non-axial arrangements. One of the defined surfaces represent the detector (11.95 mm radius), and the other represent the source of different radii in the range 2.5 to 11.95 mm as described in Fig. 1. The calculations are performed in the S–D distance range 4 to 44 mm with 4 mm step size. The calculations are performed on a single-threaded 2.66 GHz of Intel CORE 2 Duo processor with a number of histories of 10^7^ for all input files where, a total of eleven input files are considered for each S–D configuration.

### 2.3 Numerical calculation

The *α*-counting efficiency for axial and non-axial S–D configurations in the S–D distance range 4 to 44 mm are calculated by the derivation of analytical formulas. These formulas in its integral form are then solved numerically using Simpson's rule with the aid of a designed computer code. The code is written using a C programming language and the calculation processes are performed on a 2.66 GHz processor by dividing the integration limits for each of polar *θ* and azimuthal *ϕ* angles, and the disk source radius $R_{s}$ into a 100 interval. The method calculation error is always less than 1%.

## 3 Experimental measurements

The experimental data used to benchmark the proposed method are obtained using the net count rate of *α*-standard sources located axially and ⊥ to the symmetry axis of a *α*-spectrometer.

The standard sources of radionuclides Am241, Th228, and Th230 electroplated onto Platinum Surface, are permanently fixed in an aluminum holder of type A-1 with 1″ diameter × 0.125″ high (25.4 mm × 3.18 mm) and the active diameter is 0.197″ (5.0 mm) [24]. Where, Table 1 presents the utilized *α*-standard sources specification including, their half-life, significant energies with their relative abundance, activity, and the production date.

**Table 1:** *α*-standard sources specification.

Isotope	Half-Life (Year)	*α*-Energy (keV)	$I_{\alpha }$ (%)	Activity ± error (kBq ± %)	Production date
Am-241	432.20	5388	01.6	3.635 ± 3.0	Mar. 1, 2017
		5443	13.0		
		5485	84.5		
Th-228	1.9116	5340	27.2	0.3108 ± 3.1	
		5423	72.2		
Th-230	7.5*E*04	4621	23.4	0.3642 ± 3.1	
		4687	76.3		

The ORTEC *α*-spectrometer Model (U-020-450-AS) is equipped with an ULTRA-AS Passivated Implanted Planar Silicon (PIPS) detector assembled inside a high-quality vacuum chamber with B-mounting configuration. The chamber is provided with a sample holder to adjust the S–D distances in the range 4–44 mm with 4 mm apart. The detector model is ENS-U450 with a $450\;{\text{mm}}^{2}$ active area, 100 μm depletion depth, +50 V applied bias voltage, and a 20 keV resolution (FWHM) at the 5486 keV *α*-energy line of ${\text{Am}}^{241}$. The detector operates in the energy range 0 to 10 MeV with a Multi-Channel Analyzer (MCA) of 2,048 channels. The spectrometer is controlled using the acquisition and data analysis software (MAESTRO-32 [25], and Alpha Vision-32 [26]).

Experimental measurements are performed using the aforementioned *α*-standard sources with axial configuration and for S–D distances range 7 to 39 mm from the front facet of an *α*-detector with step size of 4 mm. For all the measurements, the dead time is below 0.5%, and the acquisition time is adjusted in such away that the counting statistics in the measured peaks is below 1%.

## 4 Results and discussion

In this work, formulas for a symmetric and an axisymmetric S–D configurations are derived to calculate the counting efficiency of an *α*-detector (11.95 mm radius, and $450\;{\text{mm}}^{2}$ active area) mathematically. The formulas in their integral form are solved numerically using a written C computer code based on Simpson's integration method. The calculations are carried out for different disk source radii range 2.5 to 11.95 mm (less than and equal the detector radius) and for S–D distances range 4 to 44 mm in case of the axial S–D configuration. While, a disk source of radius 5 mm parallel to the detector and at lateral distances in the range of 2 to 5 mm and at a maximum S–D distance of 44 mm is used for detector efficiency in the non-axial S–D configuration case. The aforementioned numerical calculations are carried out by dividing the integration limits for the polar *θ*, azimuthal *ϕ* and the source radius into a 100 interval. The calculation is performed on a single-threaded 2.66 GHz of Intel CORE 2 Duo processor and the calculation time is always less than 1 ms, while the relative standard deviation is less than 1%. In order to benchmark the derived integral formulas, the MCNPX program is used to simulate the configurations mentioned above and calculation are carried out on the same computer machine with number of histories of 10^7^ for a total of 99 input files with 1.5 min calculation time for each and less than 1% relative standard deviation. Table 2 presents the counting efficiency of both MCNP and the proposed method for axial S–D configuration while, Table 3 illustrate the relative difference between the two methods. The results show an agreement between the two methods with a maximum relative difference of about 0.2%. Also, Table 4 presents the counting efficiency of the two methods in the non-axial S–D configuration with their relative standard deviation. The used method accuracy is of 0.1% with respect to the MCNP calculation.

**Table 2:** *α*-counting efficiency for parallel and axial S–D configuration vs. S–D distance (*h*) and different disk source radii *R*_*s*_ comparison between MCNP (*ε*_*MC*_) and Numerical (*ε*_*N*_).

	Efficiency$\left(R_{s}<R_{d}\right)$						Efficiency$\left(R_{s}=R_{d}\right)$	
*R*_*s*_ (mm)	2.5		5.0		7.5		11.95	
*h* (mm)	*ε*_*MC*_	*ε*_*N*_	*ε*_*MC*_	*ε*_*N*_	*ε*_*MC*_	*ε*_*N*_	*ε*_*MC*_	*ε*_*N*_
4	0.33892	0.33914	0.33205	0.33219	0.31843	0.31859	0.26600	0.26602
8	0.21951	0.21966	0.21280	0.21297	0.20139	0.20152	0.16983	0.16986
12	0.14417	0.14427	0.13989	0.14000	0.13295	0.13308	0.11544	0.11558
16	0.09850	0.09856	0.09607	0.09609	0.09209	0.09215	0.08224	0.08234
20	0.07028	0.07030	0.06884	0.06887	0.06660	0.06659	0.06079	0.06089
24	0.05216	0.05212	0.05131	0.05128	0.04992	0.04991	0.04642	0.04645
28	0.04001	0.03995	0.03949	0.03943	0.03859	0.03858	0.03633	0.03639
32	0.03155	0.03148	0.03120	0.03114	0.03063	0.03059	0.02911	0.02916
36	0.02544	0.02539	0.02521	0.02516	0.02480	0.02479	0.02380	0.02382
40	0.02089	0.02087	0.02073	0.02071	0.02045	0.02046	0.01978	0.01978
44	0.01747	0.01744	0.01732	0.01733	0.01714	0.01715	0.01667	0.01667

**Table 3:** The relative standard deviation of the calculated efficiency using MCNP (*ε*_*MC*_) and numerical (*ε*_*N*_) for parallel and axial S–D configuration vs. S–D distance (*h*) at different disk source radii (*R*_*s*_).

	$Deviation\text{\%}=(\frac{\varepsilon_{MC}-\varepsilon_{N}}{\varepsilon_{MC}})⁎100$				
*R*_*s*_ (mm)	2.5	5.0	7.5	10	11.95
*h* (mm)					
4	-0.065	-0.041	-0.051	-0.028	-0.007
8	-0.070	-0.082	-0.065	0.003	-0.016
12	-0.071	-0.081	-0.096	-0.070	-0.128
16	-0.061	-0.028	-0.070	-0.017	-0.129
20	-0.025	-0.043	0.009	-0.093	-0.166
24	0.069	0.069	0.020	-0.022	-0.083
28	0.147	0.174	0.042	-0.105	-0.177
32	0.217	0.178	0.130	-0.120	-0.186
36	0.227	0.194	0.039	-0.070	-0.090
40	0.112	0.096	-0.020	-0.036	-0.030
44	0.169	-0.051	-0.030	0.002	0.036

**Table 4:** *α*-counting efficiency for parallel and axi-symmetric S–D configuration with lateral distance *ρ* vs. S–D distance (*h*) comparison between MCNP (*ε*_*MC*_) and Numerical (*ε*_*N*_) with their relative standard deviation, the disk source radius *R*_*s*_ = 5 mm.

*ρ* (mm)	2.0			4.0			6.95		
*h*(mm)	*ε*_*MC*_	*ε*_*N*_	*RSD* (%)	*ε*_*MC*_	*ε*_*N*_	*RSD* (%)	*ε*_*MC*_	*ε*_*N*_	*RSD* (%)
4	0.32872	0.32850	0.068	0.31743	0.31726	0.054	0.28046	0.28055	-0.032
8	0.21004	0.20987	0.077	0.20118	0.20099	0.096	0.17729	0.17743	-0.077
12	0.13823	0.13814	0.069	0.13300	0.13286	0.102	0.11955	0.11956	-0.044
16	0.09509	0.09507	0.017	0.09212	0.09216	-0.040	0.08459	0.08458	0.016
20	0.06829	0.06831	-0.027	0.06657	0.06660	-0.039	0.06233	0.06219	0.223
24	0.05093	0.05096	-0.069	0.04990	0.04992	-0.034	0.04727	0.04725	0.054
28	0.03921	0.03923	-0.055	0.03857	0.03860	-0.079	0.03692	0.03690	0.078
32	0.03100	0.03103	-0.098	0.03058	0.03058	0.000	0.02949	0.02949	0.007
36	0.02506	0.02511	-0.182	0.02478	0.02481	-0.098	0.02402	0.02404	-0.110
40	0.02065	0.02069	-0.182	0.02045	0.02048	-0.137	0.01993	0.01994	-0.037
44	0.01728	0.01731	-0.151	0.01715	0.01716	-0.103	0.01680	0.01678	0.161

**Figure 2:**
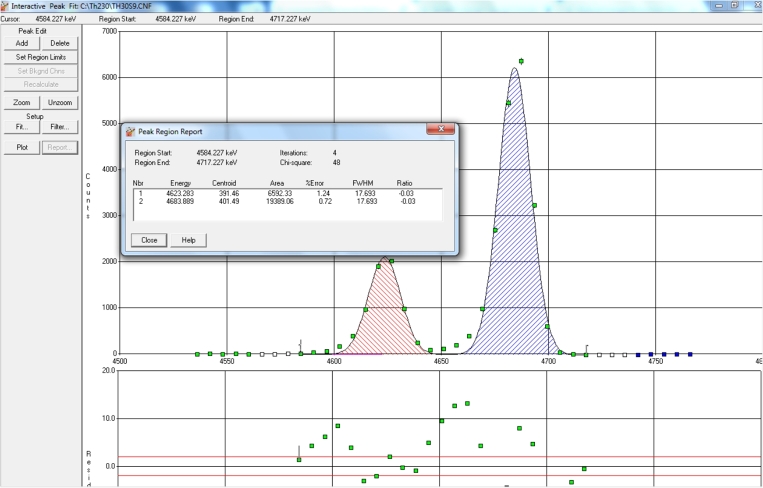
Peak area acquisition using the interactive peak fit.

In order to benchmark the proposed calibration method against experimental measurements. The count rates of three *α*-emitter disk shaped sources (Am-241, Th-228, and Th-230) are measured axially and at S–D distances in the range 7–39 mm while the count rate error is always less than 2%. The spectrums are acquainted using Mastero 32 software, while the spectrum analysis is carried out using Alpha Analysis Software (Genie 2000) [27], with the corresponding alpha library of each isotope. Where, analysis sequence of peak locate using unidentified 2nd order differential, peak locate using sum/Non linear square fit, and interactive peak fit without filters [27], [28], [29] and the spectrum acquisition time are used to obtain the net count rate for the desired peak as shown in Fig. 2.

Once the count rates are acquainted for each isotope, efficiency calibration is carried out using the proposed method and the source activity is calculated using Equation (1). The estimated activities using the proposed method are then compared to the certified values and the relative standard deviation is calculated as presented in Table 5. The estimated activities using the proposed numerical method show an agreement with the certified activity values with accuracy of 0.16 to 1.65%. Accordingly the self-attenuation, scattering effect, and the detector edge effect contributions for the employed standard sources is less than 1.7%.

**Table 5:** The relative standard deviation of the calculated Activity using the proposed numerical method (*A*_*N*_) and the certified corrected Activity (*A*_*c*_) for parallel and axial S–D configuration.

	$Deviation\;\text{\%}=(\frac{A_{N}-A_{C}}{A_{C}})\times 100$		
h(mm)	Am-241	Th-228	Th-230
7	1.65	0.10	-1.10
11	-1.76	1.10	-0.35
15	-1.61	1.31	-0.66
19	-1.38	-1.37	0.70
23	-1.30	-0.21	1.14
27	-1.27	-1.42	0.77
31	-0.62	1.46	-0.19
35	-0.56	0.79	-0.16
39		1.59	0.78

### 4.1 Limitation of the proposed method and future work

**Figure 3:**
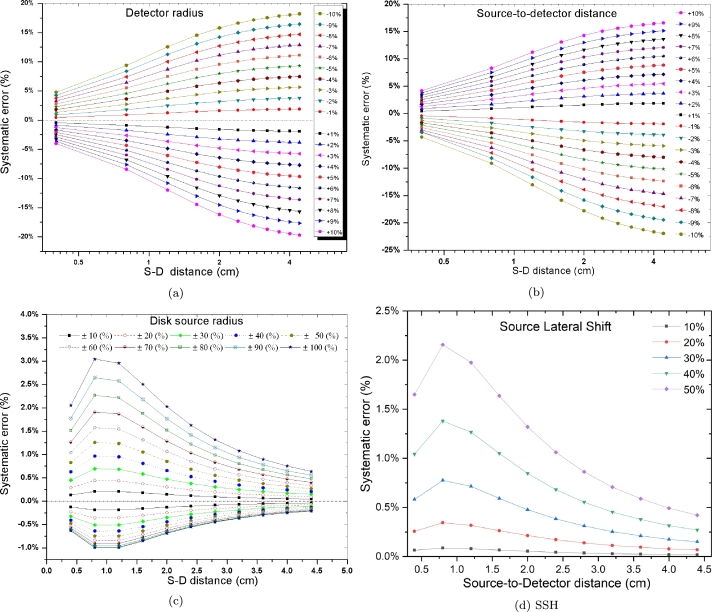
Percentage systematic error introduced on the calculated efficiency for axial disk source-to-detector configuration by in accurate values; (a) detector radius, (b) source-to-detector distance, (c) disk source active radius, and (d) source-to-detector lateral distance.

The usage of data such as the detector's dimensions (height, and radius), S–D configuration, and distance could lead to significant criticism of the proposed method. These parameters are frequently believed to be underspecified. The uncertainty on these parameters could lead to either large, small, or has no effect on the final ratio of solid angle values for an *α*-spectrometer in question (and thus of the efficiency). It appears necessary to investigate whether any significant ambiguity in the above-mentioned parameters makes the method's use questionable. Wherefore, solid angle values for various energies were calculated using parameters other than those initially stated, as follows:

±10% of the original *α*-detector radius, and the S–D distance with a 1% step, ±100% of the disk source radius with 10% step, and 50% for lateral disk shaped source with respect to its radius with 10% step. This is conducted at various S–D distances, through using efficiency calculated for a disk (2.5 mm radius)-to-detector (11.95 mm radius) setup at various distances in the range (4 to 44 mm) as a reference. The *α*-detector efficiency vs. S–D distance were calculated using Eqn. (5) numerically for the aforementioned inaccurate values. After that, the efficiency results are compared to the efficiency data acquired with the original parameters data. The results for the aforementioned parameters are presented in Fig. 3. It is obvious that, in the considered arrangement the results referring to that the data pertaining to the detector radius and S–D distance are highly critical, where inaccuracy on these parameters by ±1% can cause inaccuracy in the results by around ±2% as illustrated in Fig. 3-a and b, so due care has to be taken while measuring and introducing these parameters using the presented method.

Furthermore, it is revealed that imprecise source radius, lateral distance data have a very minor effect on the computed efficiency at various S–D distances. Fig. 3-c and d shows that ±50% inaccuracy in the source radius, lateral distance can, yields only less than ±3% uncertainty on the calculated efficiency. Nevertheless, accurate determination of these parameters would improve the accuracy of the data. Therefore, the optimum condition while using absolute calibration techniques is to measure (specify) the detector radius and S–D distance with no more than ±1% accuracy, but for the source diameter, and lateral distance even ±10% accuracy would lead to less than ±0.5% inaccuracy of the final results.

Other parameters including the detector thickness, and dead layer, source active thickness, and their backing material (thickness and type), and the measuring environment that cause inaccuracies in the measuring activities due to self attenuation, and backscattering will be discussed in the future work.

## 5 Conclusion

In this paper, we propose a numerical method for efficiency calibration of an *α*-spectrometer. Where, analytical formula for efficiency calibration of different source-to-detector configurations is derived, benchmarked, and applied for the activity calculation of various *α*-emitters. The derived formula in its integral from is solved numerically based on the Simpson's integration method, using a written C computer code to calculate the detector efficiency. To benchmark the proposed method, the MCNP code is then used to calculate the detector efficiency at different S–D configurations. An agreement between the results is found to be within 0.5%. In addition, experimental measurements of standard *α*-emitter activities are carried out based on the proposed numerical method. The estimated activities for the reference sources are found to be in agreement with the certified data. Furthermore, the inaccuracy due to experimental setup specifications is investigated using in accurate inputs (e.g. detector, and source radii, and source-to-detector distance). Results show that, the detector radius and S–D distance are very sensitive parameters for the time being. So that, detector manufacturers should pay the utmost attention during the measurement of the detector radius. The proposed method is very simple, accurate, time saving, and free of standard but for better results. It is necessary that the experimental setup specifications be accurately characterized, and that the S–D distance be measured with an accuracy of less than 0.1 mm. In fact, this method is an absolute numerical method that overcome the anomalies in relative calibration methods using standard calibration sources. It could be used efficiently in the field of NM characterization and verification using *α*-spectrometry. This method could be extended to include the source self-attenuation and the *α*-backscattering factors.

## Additional information

No additional information is available for this paper.

## CRediT authorship contribution statement

**K.M. El-Kourghly:** Writing – review & editing, Writing – original draft, Visualization, Validation, Software, Project administration, Methodology, Investigation, Formal analysis, Data curation, Conceptualization. **W. El-Gammal:** Supervision, Conceptualization, Writing – review & editing, Formal analysis, Project administration. **Mohamed M. Damoom:** Conceptualization, Supervision, Writing – original draft, Writing – review & editing. **Abdulsalam M. Alhawsawi:** Supervision, Resources, Funding acquisition. **M. Abdelati:** Writing – original draft, Supervision, Data curation. **H.I. Khedr:** Supervision, Resources. **R. Abouzaid:** Data curation, Writing – original draft.

## Declaration of Competing Interest

The authors declare that they have no known competing financial interests or personal relationships that could have appeared to influence the work reported in this paper.

## Figures and Tables

**Table:** 

No	Configuration	*ρ*	*θ*	*ϕ*
1	(*h* > 0&*R*_*s*_ < *R*_*d*_) (l=0)	*ρ*_1,1_(1)=*ρ*_0_	*θ*_1,1_(1)=*θ*_0_	*ϕ*_1,1_(1)=*ϕ*_0_
		*ρ*_1,1_(2)=*ρ*_1_	*θ*_1,1_(2)=*θ*_1_	*ϕ*_1,1_(2)=*ϕ*_1_

2	(*h* > 0&*Rs* = *Rd*) (l=0)	*ρ*_2,1_(1)=*ρ*_0_	*θ*_2,1_(1)=*θ*_0_	*ϕ*_2,1_(1)=*ϕ*_0_
		*ρ*_2,1_(2)=*ρ*_1_	*θ*_2,1_(2)=*θ*_1_	*ϕ*_2,1_(2)=*ϕ*_1_
		*ρ*_2,2_(1)=*ρ*_0_	*θ*_2,2_(1)=*θ*_0_	*ϕ*_2,2_(1)=*ϕ*_0_
		*ρ*_2,2_(2)=*ρ*_1_	*θ*_2,2_((2)=*θ*_1_	*ϕ*_2,2_(2)=*ϕ*_2_

3	(*h* > 0&*R*_*s*_ + *l* < *R*_*d*_)	*ρ*_3,1_(1)=*ρ*_0_	*θ*_3,1_(1)=*θ*_0_	*ϕ*_3,1_(1)=*ϕ*_0_
		*ρ*_3,1_(2)=*ρ*_2_	*θ*_3,1_(2)=*θ*_1_	*ϕ*_3,1_(2)=*ϕ*_1_

4	(*h* > 0&*R*_*s*_ + *l* = *R*_*d*_)	*ρ*_4,1_(1)=*ρ*_0_	*θ*_4,1_(1)=*θ*_0_	*ϕ*_4,1_(1)=*ϕ*_0_
		*ρ*_4,1_(2)=*ρ*_2_	*θ*_4,1_(2)=*θ*_1_	*ϕ*_4,1_(2)=*ϕ*_1_
		*ρ*_4,2_(1)=*ρ*_0_	*θ*_4,2_(1)=*θ*_0_	*ϕ*_4,2_(1)=*ϕ*_0_
		*ρ*_4,2_(2)=*ρ*_2_	*θ*_4,2_(2)=*θ*_1_	*ϕ*_4,2_(2)=*ϕ*_2_

$\rho_{0}=\theta_{0}=\phi_{0}=0$				
$\phi_{1}=\pi$				
$\phi_{2}=\pi /2$				
$\rho_{1}=R_{s}$				
$\rho_{2}=-l\cos\phi +\sqrt{R_{s}^{2}-l^{2}\sin^{2}\phi }$				
$\theta_{1}=\tan^{-1}(\frac{\rho_{1}\cos\phi +\sqrt{R_{d}^{2}-\rho^{2}\sin^{2}\phi }}{h})$				
$\theta_{2}=\tan^{-1}(\frac{\rho_{2}\cos\phi +\sqrt{R_{d}^{2}-\rho^{2}\sin^{2}\phi }}{h})$				

**Figure 4:**
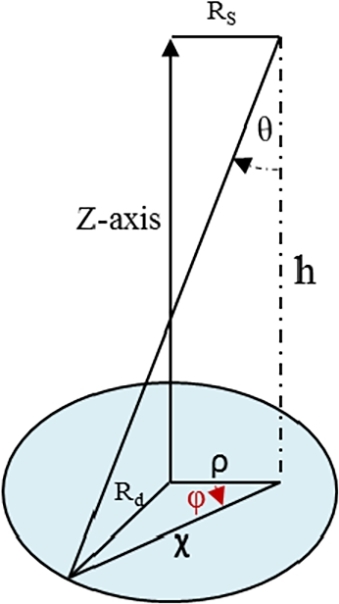
Geometrical illustration of the approach used to derive the polar angle for non-axial point.

## Data Availability

The raw/processed data necessary to reproduce the aforementioned findings have been included as part of the current study.

## Appendix B Integration limits for polar angles

As illustrated in Fig. 4, the polar angle *θ* could be given by,

(6) $$\theta =\tan^{-1}(\chi /h).$$

Using the cosine rule we get,

(7) $$R_{d}^{2}=\chi^{2}+\rho^{2}-2\;\chi \;\rho \;\cos\phi .$$

By rearranging Eqn. (7) we get the following formula,

(8) $$\chi^{2}-2\rho \cos\phi \;\chi +\rho^{2}-R_{d}^{2}=0;$$

Equation (8) represents a form a quadratic equation, where a=1, b=−2ρcos⁡ϕ are the coefficients, *χ* is the variable, and $c=\rho^{2}-R_{d}^{2}$ is the constant term. Using the quadratic formula to solve Equation (8) we get the following form,

(9) $$\chi =\rho \cos\phi \pm \sqrt{R_{d}^{2}-\rho^{2}\sin^{2}\phi };$$

knowing that $\rho <R_{d}$ thus, Equation (9) could take the following form,

(10) $$\chi =\rho \cos\phi +\sqrt{R_{d}^{2}-\rho^{2}\sin^{2}\phi }.$$

Thus, the polar angle *θ* could be given using Equation (10) into Equation (6) where,

(11) $$\theta =\tan^{-1}(\frac{\rho \cos\phi +\sqrt{R_{d}^{2}-\rho^{2}\sin^{2}\phi }}{h}).$$
